# Jia-Wei-Yu-Ping-Feng-San Attenuates Group 2 Innate Lymphoid Cell-Mediated Airway Inflammation in Allergic Asthma

**DOI:** 10.3389/fphar.2021.703724

**Published:** 2021-07-09

**Authors:** Lingna Xue, Cui Li, Guangbo Ge, Shaoyan Zhang, Liming Tian, Yu Wang, Huiyong Zhang, Zifeng Ma, Zhenhui Lu

**Affiliations:** ^1^ Institute of Respiratory Disease, Longhua Hospital Shanghai University of Traditional Chinese Medicine, Shanghai, China; ^2^ Institute of Interdisciplinary Integrative Medicine Research, Shanghai University of Traditional Chinese Medicine, Shanghai, China

**Keywords:** Jia-Wei-Yu-Ping-Feng-San, allergic asthma, airway inflammation, group 2 innate lymphoid cells, traditional Chinese medicine

## Abstract

The incidence of asthma has increased in recent decades. Although corticosteroids and bronchodilators are used in clinical practice, the control of asthma remains a challenge. Allergic asthma is characterized airway inflammation mediated by type 2 immune response. Group 2 innate lymphoid cells (ILC2s) are an important source of type 2 cytokines IL-5 and IL-13, which contribute to the progress of asthma. Jia-Wei-Yu-Ping-Feng-San (JWYPFS), a traditional Chinese medicine, has been widely used to treat asthma in China. In this study we investigated the mechanisms of JWYPFS in the treatment of asthma, especially the effect on ILC2s important in airway inflammation. Female C57BL/6 mice were sensitized and challenged with OVA to establish a model of allergic asthma. Airway hyperresponsiveness was examined by direct airway resistance analysis. Inflammatory cell counts were determined in bronchoalveolar lavage fluid (BALF). Inflammatory cell infiltration and mucus hypersecretion in lung tissue sections was observed by HE and PAS staining, respectively. The numbers and proportions of ILC2s as well as the ILC2s-related transcription factors GATA3, IRF4, and type 2 cytokines were measured in lung tissue samples. Additionally, ILC2s were collected from mouse lung; ILC2s-related cytokines and GATA3 and IRF4 were evaluated after IL-33-induced activation of ILC2s *in vitro*. Elevated inflammatory cells, mucus secretion, airway hyperresponsiveness and type 2 cytokines in the OVA-treated asthma group indicated that an allergic asthma model had been established. JWYPFS treatment attenuated airway resistance and reduced inflammatory cells including eosinophils, and inhibited mucus production and type 2 cytokines in these asthmatic mice. Moreover, JWYPFS treatment dramatically decreased the numbers and proportions of ILC2s and the mRNA levels of GATA3 and IRF4. In an *in vitro* experiment JWYPFS significantly suppressed GATA3, IRF4 and type 2 cytokine expression, including IL-5 and IL-13 in IL-33-stimulated ILC2s. JWYPFS alleviates ILC2s-mediated airway inflammation, suggesting that JWYPFS might be an effective agent to treat allergic asthma.

## Introduction

Asthma is a common chronic inflammatory disease of the airways. It affects approximately 1–18% of the population in different countries, and about 350 million people suffer from asthma worldwide (Ish et al., 2020). In China, the prevalence of asthma is 4.2%, representing 45.7 million Chinese adults (Huang et al., 2019). Asthma causes a heavy burden to governments, families and patients (Loftus and Wise, 2015).

The pathogenetic mechanisms of asthma are extremely complicated. Many previous studies have indicated that T helper 2 (Th2) cells play a crucial role in the progression of the asthma disease process (Wills-Karp et al., 1998; Kips et al., 2001; Choy et al., 2015). After contact with the antigen or allergen presented by antigen-presenting cells (such as dendritic cells), Th2 produces interleukin (IL)-5, IL-13 and other type 2 cytokines, and then causes eosinophil (Eos) infiltration into the airways. However, the discovery of innate lymphoid cells (ILCs) has changed our understanding of the pathogenesis of asthma over the last decade.

Unlike Th2 cells, ILCs are a newly found class of lymphocytes lacking specific antigen receptors. Based on the expression of transcription factors and effector cytokines, ILCs can be divided into the following classes: ILC1s, ILC2s, and ILC3s (Wang et al., 2017; Vivier et al., 2018). Both Th2 cells and ILC2s contribute to eosinophilic airway inflammation in allergic asthma (Li et al., 2019). Epithelial cells can be activated directly by the allergen and then release cytokines, such as thymic stromal lymphopoietin (TSLP), IL-25 and IL-33. These cytokines caused further activation of ILC2s to produce type 2 cytokines such as IL-5 and IL-13. Animal models and studies on humans have confirmed that ILC2s have a significant effect on allergic asthma, especially on difficult-to-treat and severe asthma (Smith et al., 2016; Lund et al., 2017). Inhaled corticosteroids (ICS), beta2-agonists and bronchodilators are the main drugs recommended by the current Global Initiative for Asthma guidelines for the treatment of asthma (Muneswarao et al., 2019). However, the effectiveness of these drugs varied between patients. Adverse effects, corticosteroid-insensitivity, and steroid resistant are long existing problems for patients and clinicians. Therefore, it is urgent and necessary to establish a more complete, individualized and precise treatment strategy.

Traditional Chinese medicine (TCM) treatment of asthma has significant clinical efficacy and few adverse effects, and is well received by patients in China (Jiang et al., 2018; Ma et al., 2018). Yupingfeng San (YPFS) originates from Danxi Xinfa by Zhu Danxi of the Yuan dynasty, and it is a combination of *Astragalus mongholicus* Bunge, *Atractylodes macrocephala* Koidz. and *Saposhnikovia divaricata* (Turcz. ex Ledeb.) Schischk. Its efficacy is to invigorating qi and consolidation of superficies. The effectiveness of YPFS is satisfactory with asthma and has few adverse reactions (Chen et al., 2014; Wang et al., 2020). It was found that YPFS has anti-allergic effects and can inhibit the production of IgE (Bao et al., 2019; Wang et al., 2020). YPFS can effectively suppress the Th2 response and regulate the balance of T cell subsets (Wang et al., 2016). Jia-Wei-Yu-Ping-Feng-San (JWYPFS) is modified from YPFS with the addition and subtraction rules of TCM. According to the theories of TCM, the pathogenesis of asthma has a relationship with phlegm and “Yang” deficiency. *Cullen corylifolium* (L.) Medik., *Aconitum carmichaeli* Debeaux, *Schisandra chinensis* (Turcz.) Baill and *Rubus chingii* Hu are included to enhance Yang, resist chills and reduce phlegm. *Vitex negundo* L., *Glycyrrhiza uralensis* Fisch. ex DC., and *Imperata cylindrica* (L.) P. Beauv. are added to *clear fever and decrease phlegm*. JWYPFS and related components have been widely used in the treatment of respiratory diseases, such as respiratory viral infections, allergic rhinitis and so on. The mechanism of JWYPFS effects may be regulating T cells homeostasis or reduced the susceptibility of cells to the invasion of human respiratory syncytial virus by inhibiting the expression of intercellular cell adhesion molecule-1 (Liu et al., 2013; Chan and Chien, 2014). However, the underlying mechanism of JWYPFS on asthma is not clear. Therefore, the purpose of this study was to investigate the effects of JWYPFS on airway inflammation in allergic asthma, especially the type 2 inflammatory response caused by ILC2s.

## Materials and Methods

### Animals and Reagents

Female C57BL/6 mice (aged 6–8 weeks) were supplied by Vital River Laboratory Animal Technology Co. Ltd. (Beijing, China) and housed in a pathogen-free rodent facility with free access to water and food. This study was performed at the Laboratory Animal Center of Longhua Hospital Shanghai University of Traditional Chinese Medicine (Shanghai, China) in accordance with the Care and Use of Laboratory Animals (NIH publication 86-23, revised 1985). The animal experiment was approved by the Institutional Animals Care and Use Committee (IACUC) of Longhua Hospital Shanghai University of Traditional Chinese Medicine (No. 2019-N032).

Ovalbumin (OVA, grade V, A5503) and DNase I were purchased from Sigma Chemical Co. (St. Louis, MO, United States) and Imject alum was purchased from Thermo Scientific. Dexamethasone sodium phosphate injection (5 mg/ml) was obtained from Xianju Medicine Co. (Zhejiang, China). ELISA kits for IL-5 and IL-13 were supplied by Multisciences (Lianke) Biotech, Co., LTD (Hangzhou, China). Liberase TM was purchased from Roche Diagnostics Corporation (Indianapolis, IN, United States). FITC-labeled anti-mouse Lineage Cocktail, APC-labeled anti-mouse KLRG1, PE-labeled anti-mouse Sca-1, PE-Cy7-labeled anti-mouse IL-13 and Fixable Viability Dye eFluor 780 were provide by eBioscience Inc. (San Diego, CA, United States). BV421-labeled anti-mouse/human IL-5, AF700-labeled anti-mouse CD45, and recombinant mouse IL-33 were obtained from Biolegend (CA, United States).

The following natural compounds were provided by Shanghai Standard Technology Co., Ltd.(Shanghai, China): Astragaloside II (Lot No. 3824), Psoralenoside (Lot No.7816), Tigloylgomisin H (Lot No. 8549), Liquiritin apioside (Lot No. 6797), Vitexin (Lot No. 3593), Calycosin (Lot No. 7851), Liquiritigenin (Lot No. 4280), Isopsoralenoside (Lot No. 7608), Orientin (Lot No. 2920) and Schisandrol B (Lot No. 9165). The following standard reference compounds were obtained from Chengdu Must Bio-technology Co., Ltd. (Sichuan, China): Ellagic Acid (Lot No. MUST-20111705), Schisandrol A (Lot No. MUST-20031905), Glycyrrhizic acid (Lot No. MUST-20122305), Astragaloside IV (MUST-20090308), 4′-O-β-Glucopyranosyl-5-O-methylvisamminol (Lot No. MUST-20050410), Liquiritin (Lot No. MUST-20052110), Benzoylhypaconine (Lot No. MUST-20033111), Hypaconitine (Lot No. MUST-20052710), Atractylenolide I (Lot No. MUST-20101101), Prim-O-glucosylcimifugin (MUST-20022711) and Benzoylmesaconine (Lot No. MUST-20022710). The purities of these compounds were over 98%.

### Preparation of the Herbal Medicine

The herbal medicine (JWYPFS granules) is composed of ten individual herbs. Medicinal herbs are extracted, concentrated, dried, and processed into granules. The granules of each herb can be purchased by Chinese hospitals. All herbal granules used in this study were provided by Sichuan Neo-Green Pharmaceutical Technology Development Co., Ltd. (component granules and batch number are shown in Table 1). In this study, the granules of ten individual herbs were obtained from Longhua hospital and mixed according to the dose recommended by the doctors. Briefly, JWYPFS granules were dissolved in sterile water and the final concentrations used for oral administration were 1.0, 2.0, and 4.0 g/kg body weight. The dose of JWYPFS administered to mice was converted according to human equivalent dose based on body surface area.

**TABLE 1:** Composition and doses of JWYPFS.

Chinese name	Botanical plant name	Botanic family	English name	Amount (g)	Batch number
Huang qi	*Astragalus mongholicus* Bunge	Fabaceae Lindl	Milkvetch root	20	19090056
Bai Zhu	*Atractylodes macrocephala* Koidz	Asteraceae Bercht. and J. Presl	Largehead Atractylodes Rhizome	10	19010235
Fang Feng	*Saposhnikovia divaricata* (Turcz. ex Ledeb.) schischk	Apiaceae Lindl	Divaricate Saposhnikovia Root	6	19091048
Gan Cao	*Glycyrrhiza uralensis* Fisch. ex DC.	Fabaceae Lindl	Liquoric Root	4	19100008
Wu Wei Zi	*Schisandra chinensis* (Turcz.) Baill	Schisandraceae Blume	Chinese Magnoliavine Fruit	6	19030071
Fu Pen Zi	*Rubus chingii* Hu	Rosaceae Juss	Palmleaf Raspberry Fruit	8	19010162
Bu Gu Zhi	*Cullen corylifolium* (L.) Medik	Fabaceae Lindl	Malaytea scurfpea Fruit	10	19080180
Shu Fu Zi	*Aconitum carmichaeli* Debeaux	Ranunculaceae Juss	Prepared Common Monkshood Daughter Root	8	19020029
Huang Jing Zi	*Vitex negundo* L	Lamiaceae Martinov	Negundo Chastetree Fruit	16	18050101
Bai Mao Gen	*Imperata cylindrica* (L.) P.Beauv	Poaceae Barnhart	Lalang Grass Rhizome	20	19040078

### Chemical Profiling of JWYPFS Granules by Using LC-Q-TOF-MS

About 0.5 g of the JWYPFS was precisely weighed, placed in a 50 ml conical flask to which was added 15 ml methanol, followed by extraction with ultrasonication for 30 min, cooled to room temperature, and subjected to centrifugation at 12,000 rpm for 5 min, and the supernatant fraction was obtained.

Chromatographic separation was performed on an UPLC-Q-TOF/MS system. The UPLC system was carried out on an Agilent 1290 LC system (Agilent Technologies Inc., Palo Alto, CA, United States) equipped with a binary pump, a thermostat-controlled column compartment, an auto sampler and a DAD detector. The cooling autosampler was set at 4°C and protected from light, and the column heater was set at 30°C. Agilent ZORBAX RRHD Eclipse XDB-C18 (2.1 × 100 mm, 1.8 μm) (Agilent, United States) was employed with the temperature set at 30°C. The mobile phase consisted of A (0.1% formic acid) and B (0.1% formic acid in acetonitrile) at a flow rate of 0.3 ml min−1 and eluted by gradient elution: 0–2 min (5% B), 2–20 min (5–30% B), 20–35 min (30–50% B), 35–42 min (50–95% B), 42–44 min (95% B), 44–46 min (5% B). The injection volume was 1 µL.

The Mass spectrometer AB Sciex Triple TOF^®^ 4,600 (AB SCIEX, Foster City, CA, United States), was connected to the UHPLC system via heated electrospray ionization and controlled by Analyst TF 1.7.1. software (AB SCIEX, Foster City, CA, United States). The spectrometer was operated in full-scan TOF-MS at m/z 50-1700 and information-dependent acquisition (IDA)MS/MS modes, with negative and positive ionization modes. The optimized parameters of mass spectrometry were: Ion Source Temperature: 500°C; Curtain Gas: 35 psi; Ion Source Gas 1 and 2: 50 psi; Ion Spray Voltage: 5000 V (positive), 4500 V (negative); Declustering Potential: 100 V (MS and MS/MS); Collision Energ 40 eV; Collision Energy Spread 20eV (MS/MS); mass range: 50–1700 m/z (MS), 50–1250 m/z (MS/MS); Ion Release Delay: 30 ms; Ion Release Width: 15 ms.

Data analysis was performed by PeakView 1.2 software (AB SCIEX, Foster City, CA, United States). The phytochemical compounds were tentatively characterized based on their retention time, mass accuracy of precursor ions, MS/MS spectra and fragmentation pathways, referring to the SCIEX Natural Products HR-MS/MS Spectra Library, standard references, and literature report.

### Induction of Asthma and Animal Studies

A total of 48 mice were randomly divided into six groups (*n* = 8) as follows: a control group, an ovalbumin (OVA) asthma model group, three JWYPFS treatment groups (low dose: 1.0 g/kg/day, middle dose: 2.0 g/kg/day, high dose: 4.0 g/kg/day), and a dexamethasone (DEX) treatment group (1.0 mg/kg/day). An OVA-induced asthma model was established referring to a previous description with minor changes (Cui et al., 2019). Mice were sensitized by intraperitoneal injections with a mixture of 50 µl of 1 mg/ml OVA (grade V; Sigma) and 50 µl of Imject alum (Thermo Scientific) on day 0 and day 7. From days 14–22, mice were challenged with 3% erosolized OVA (grade II; Sigma) solution through an ultrasonic nebulizer for 30 min every other day, followed by an intranasally instilled 50 µl dose of 1 mg/ml OVA (total 50 µg) daily on days 24, 25, and 26. One day after the last challenge, mice were killed on day 28 for analysis (Figure 2A). Mice in the control group were sensitized and challenged with saline. For the treatment groups, mice were given intragastric administration of JWYPFS or DEX once a day from day 14 for 14 consecutive days (day 14–27), 1 h before OVA challenge. For the control and OVA asthma model group, mice were given the same volume of saline by intragastric administration.

### Measurement of Airway Hyperresponsiveness to Methacholine

Airway hyperresponsiveness was measured by a Buxco’s modular and invasive system (Buxco Electronics Inc., NY, United States) on day 28. Changes in the value of pulmonary resistance (RL) represented the airway resistance. The anesthetized mice was placed on an operating table, the trachea of animal was exposed and intubated through a transverse incision. The mouse was then placed in a supine position in a body plume chamber connected to a ventilator. Airway responsiveness to increased doses of Mch (0, 3.125, 6.25, 12.5, and 25 mg/ml) was recorded.

### Collection and Analysis of BALF

To obtain bronchoalveolar lavage fluid (BALF), a self-made sterile tube was inserted in the trachea and the lung was perfused twice with 1 ml of phosphate-buffered saline (PBS). The collected BALF was separated at 500 g for 10 min at 4°C. The supernatant was stored at −80°C and the cell pellet was resuspended in 100 µl PBS for cell counts. Inflammatory cells were counted by an automated cell counter (MINDRAY BC-5300vet).

### Histological Analysis of Lung Tissue

The right lower lobe of the lung was fixed in 4% formaldehyde and then dehydrated and embedded in paraffin. Thin slices (approximately 3 um) were cut and stained with hematoxylin-eosin (HE) and periodic acid Schiff (PAS). The HE staining was used to assessed inflammation of the lung. An inflammation score was determined by a pathologist on the basis of peri-bronchial and perivascular inflammation in a blind manner using the following criteria (Tong et al., 2006): 0, normal; 1, few cells; 2, a ring of inflammatory cells one cell layer deep; 3, a ring of inflammatory cells two to four cells deep; and 4, a ring of inflammatory cells of more than four cells deep. Airway mucus production was observed by PAS staining. A semi-quantitative analysis of PAS scores was determined from 10 consecutive fields per slide with 0= <5% PAS-positive goblet cells, 1 = 5–25%, 2 = 25–50%, 3 = 50–75%, and 4= >75% staining (Tong et al., 2006; Mikhak et al., 2009). Representative structures of tissues were selected and photographed.

### Measurement of Cytokines

A supernatant of BALF and lung tissue was obtained and stored at −80°C. The concentration of IL-5, IL-13 in the supernatant of BALF and lung tissue was measured by ELISA according to the manufacturer’s protocol.

### Flow Cytometric Analysis

The left lobe of lung tissue was digested with 50 μg/ml Liberase TM (Roche) and 1 μg/ml DNase I (Sigma) for 45 min at 37°C. After obtaining a cell suspension, lineage-negative cells were stained with lineage cocktail antibodies and anti-CD45 (eBioscience), anti-Sca-1 (eBioscience) and anti-KLRG1 (eBioscience) antibodies for 30 min. ILC2s were defined as CD45^+^Lineage^−^Sca-1^+^KLRG1^+^ cells (Moro et al., 2015). After staining the surface markers, cells were fixed, permeabilized and stained for intracellular IL-5 and IL-13 by their respective antibodies. For staining Th2 cells, the following antibodies were used in cell suspension: anti-CD45 (eBioscience), anti-CD4 (eBioscience) and anti-IL-4 (eBioscience) antibodies (Wang et al., 2014). IL-5^+^Th2 cells and IL-13^+^Th2 cells can be detected by added anti-IL-5 and IL-13 antibodies.

### Real-Time Polymerase Chain Reaction

Total RNA was extracted from lung tissue samples using Trizol reagent. For ILC2s *in vitro*, RNA was extracted using the Qiagen Kit according to the manufacturer’s protocol. Then, a TAKARA Reverse Transcription System Kit was used to convert total RNA into complementary DNA. *β*-Actin was selected as an internal control. The relative expression levels of ST2, IRF4, and GATA3 mRNA were determined by quantitative fluorescence PCR. The gene-specific primers for each gene are listed in Table 2.

**TABLE 2:** Primer sequences used in quantitative PCR studies.

mRNA	Primer sequences
*β-Actin*	Forward: 5’-GGC​TGT​ATT​CCC​CTC​CAT​CG-3’
	Reverse: 5’-CCA​GTT​GGT​AAC​AAT​GCC​ATG​T-3’
*ST2*	Forward: 5’-TGA​CAC​CTT​ACA​AAA​CCC​GGA-3’
	Reverse: 5’-AGG​TCT​CTC​CCA​TAA​ATG​CAC​A-3’
*IRF4*	Forward: 5’-TCC​GAC​AGT​GGT​TGA​TCG​AC-3’
	Reverse: 5’-CCT​CAC​GAT​TGT​AGT​CCT​GCT​T-3’
*GATA3*	Forward: 5’- CTC​GGC​CAT​TCG​TAC​ATG​GAA -3’
	Reverse: 5’- GGA​TAC​CTC​TGC​ACC​GTA​GC -3’

### Preparation of JWYPFS-Medicated Serum

JWYPFS-medicated serum was prepared based on previous reports (Xu et al., 2020). Ten adult male SD rats (weight 200–250 g) were provided by Vital River Laboratory Animal Technology Co. Ltd. (Beijing, China). After 1 week of adaptive feeding, the rats were randomly divided into two groups; the normal group was given saline, while the second group was treated with JWYPFS by oral gavage (0.96 g/ml, 1 ml/100 g body weight), twice a day for seven consecutive days. Two hours after the last administration, the rats were anesthetized by intraperitoneal injection of pentobarbital sodium (30 mg/kg). Blood was collected from the heart of the rat. The blood samples were left to stand 1 h and then centrifuged at 3500 rpm for 10 min, filtered with an Ultrafiltration centrifugal tube (Millipore), heated in 56°C water bath for 30 min, and stored at −80°C.

### Isolation of ILC2s From the Lung of Mouse

For expansion of ILC2s *in vivo*, C57BL/6J mice (aged 6–8 weeks) were anesthetized and 500 ng of recombinant IL-33 (BioLegend) was instilled by intranasal administration on four consecutive days. Lung tissue was collected after euthanizing the animals. Preparation of a dissociated cell suspension and streaming surface antibodies was performed as described in *Flow Cytometric Analysis*; all operations were conducted in a sterile environment. ILC2s were sorted with the BD FACS Aria.

### Cell Cultivation and Treatment

The sorted ILC2s (80,000 cells/ml) were cultured in complete culture medium. ILC2s were treated with normal serum or different concentrations of JWYPFS-medicated serum for 12 h at 37°C with 5% CO_2_. After 12 h of culture, recombinant IL-33 (20 ng/ml; BioLegend) was added for ILC2s stimulation for 4 h. Subsequently, the cells were harvested to measure levels of IRF4, GATA3, ST2 by Real-Time PCR. IL-5 and IL-13 in the cell supernatant was measured by ELISA.

### Statistical Analysis

Data was expressed as mean ± standard deviation (SD). Statistical analysis was carried out with SPSS 17.0 software (SPSS Inc., Chicago, IL, United States). Student’s t-test or one-way analysis of variance (ANOVA) was employed for statistical analysis to compare the differences between each group. Graphs were generated using GraphPad Prism 5.0 software (GraphPad Software Inc., San Diego, CA, United States). *p* < 0.05 was considered statistically significant.

## Results

### Chemical Profiling of the Constituents in JWYPFS

**FIGURE 1:**
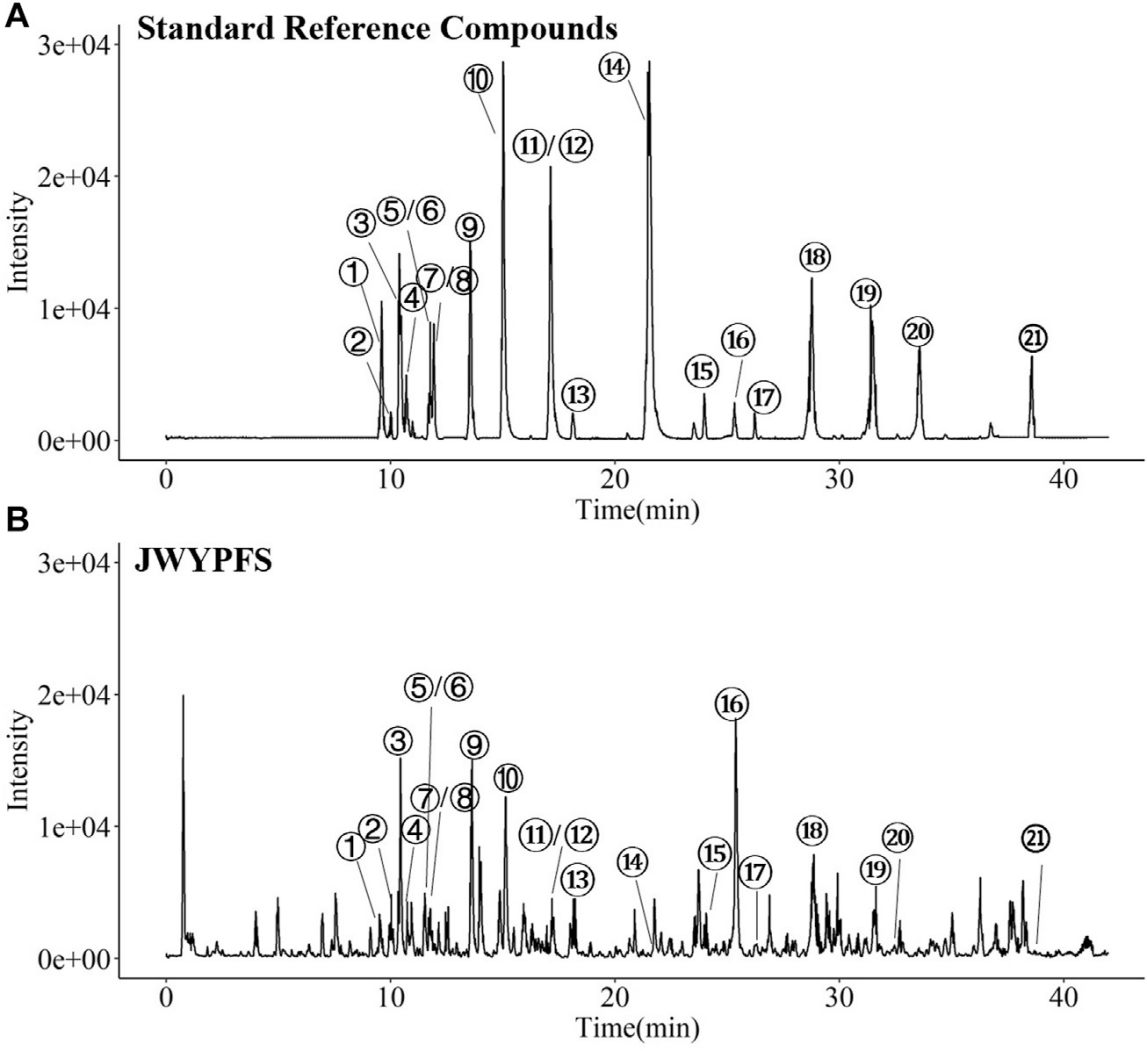
HPLC-Q-TOF-MS. Chromatographical profile of standard reference **(A)** and JWYPFS **(B)**. The main components represented by number 1–21 are shown in Table 3. ①Psoralenoside, ②Isopsoralenoside, ③Prim-O-glucosylcimifugin,④Orientin, ⑤Liquiritin, ⑥Liquiritin apioside, ⑦Vitexin, ⑧Ellagic Acid, ⑨4′-O-β-Glucopyranosyl-5-O-methylvisamminol,⑩Benzoylmesaconine, ⑪Benzoylhypaconine, ⑫Liquiritigenin, ⑬Calycosin, ⑭Hypaconitine, ⑮Astragaloside IV, ⑯Glycyrrhizic acid, ⑰Astragaloside II, ⑱Schisandrol A, ⑲Schisandrol B, ⑳Tigloylgomisin H and ㉑Atractylenolide I.

The major constituents in JWYPFS granules were analyzed by HPLC-Q-TOF-MS (Figure 1). 21 compounds are selected for LC-MS analysis, owing to these compounds are major bioactive constituents and the markers for quality control of these herbs, which are recommended by the Chinese Pharmacopeia 2020. The results show that compared with the standard reference natural compounds (Figure 1A), JWYPFS samples contained abundant chromatographic peaks (Figure 1B), indicating that there was a large number of natural constituents, and the detailed information on all detected constituents is shown in Table 3. Following comparison of the retention times and fragment ions with reference natural compounds and related databases and literature, a total of 21 constituents in JWYPFS granules were tentatively identified, including psoralenoside, isopsoralenoside, prim-*O*-glucosylcimifugin, orientin, liquiritin, liquiritin apioside, vitexin, ellagic acid, 4′-*O*-β-glucopyranosyl-5-*O*-methylvisamminol, benzoylmesaconine, benzoylhypaconine, liquiritigenin, calycosin, hypaconitine, astragaloside IV, glycyrrhizic acid, astragaloside II, schisandrol A, schisandrol B, tigloylgomisin H and atractylenolide I.

**TABLE 3:** Identification of 21 components of JWYPFS by standards and HPLC-Q-TOF-MS.

No	Compound	RT (min)	Plant Origin	No	Compound	RT (min)	Plant origin
1	Psoralenoside	10.23	*Cullen corylifolium* (L.) Medik	12	Liquiritigenin	18.09	*Glycyrrhiza uralensis* Fisch. ex DC.
2	Isopsoralenoside	10.76	*Cullen corylifolium* (L.) Medik	13	Calycosin	19.04	*Astragalus mongholicus* Bunge
3	Prim-O-glucosylcimifugin	11.09	*Saposhnikovia divaricata* (Turcz. ex Ledeb.) schischk	14	Hypaconitine	22.58	*Aconitum carmichaeli* Debeaux
4	Orientin	11.49	*Vitex negundo* L.*/Imperata cylindrica* (L.) P.Beauv	15	Astragaloside IV	24.93	*Astragalus mongholicus* Bunge
5	Liquiritin	12.49	*Glycyrrhiza uralensis* Fisch. ex DC.	16	Glycyrrhizic acid	26.36	*Glycyrrhiza uralensis* Fisch. ex DC.
6	Liquiritin apioside	12.52	*Glycyrrhiza uralensis* Fisch. ex DC.	17	Astragaloside II	27.25	*Astragalus mongholicus* Bunge
7	Vitexin	12.61	*Vitex negundo* L	18	Schisandrol A	29.67	*Schisandra chinensis* (Turcz.) Baill
8	Ellagic acid	12.69	*Rubus chingii* Hu	19	Schisandrol B	32.48	*Schisandra chinensis* (Turcz.) Baill
9	4′-O-β-Glucopyranosyl-5-O-methylvisamminol	14.29	*Saposhnikovia divaricata* (Turcz. ex Ledeb.) schischk	20	Tigloylgomisin H	34.54	*Schisandra chinensis* (Turcz.) Baill
10	Benzoylmesaconine	15.89	*Aconitum carmichaeli* Debeaux	21	Atractylenolide I	41.65	*Atractylodes macrocephala* Koidz
11	Benzoylhypaconine	18.05	*Aconitum carmichaeli* Debeaux	-			

### JWYPFS Attenuated OVA-Induced Airway Inflammation as Measured in BALF

**FIGURE 2:**
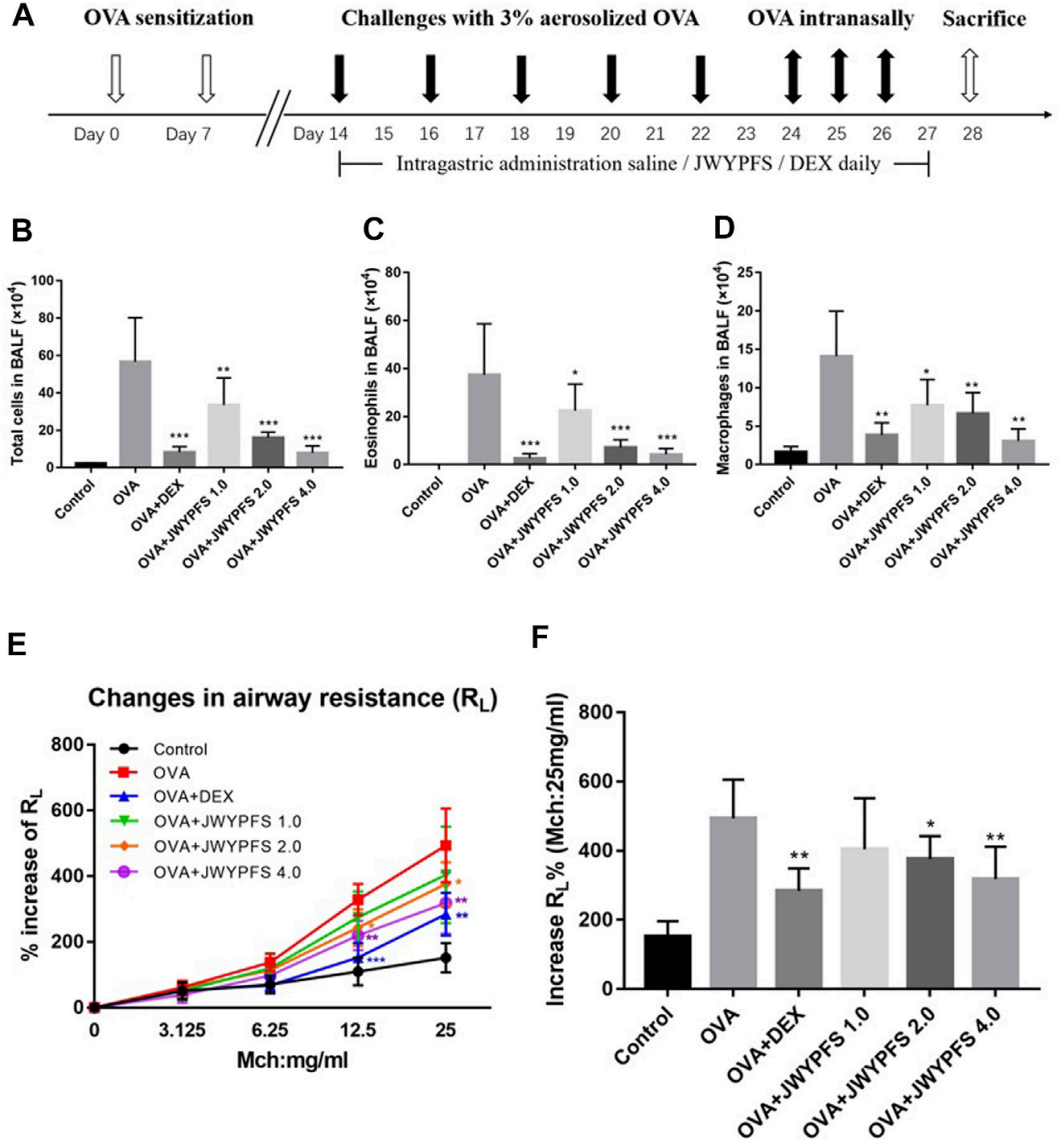
The effect of JWYPFS on inflammatory cells in BALF and on airway resistance. **(A)** Schematic diagram of the establishment of an asthma model. **(B)** Total cells in BALF. **(C)** Eos in BALF. **(D)** Macrophages in BALF. **(E)** Effect of JWYPFS on the increase of R_L_% to methacholine; **(F)** increase of R_L_% (Mch:25 mg/ml). Data are shown as mean ± SD. *p < 0.05, **p < 0.01, ***p < 0.001, compared with OVA group.

To assess airway inflammation in asthmatic mice, animals were euthanized to collect BALF on day 28. Compared with the control mice, the total cell count in the OVA group increased significantly. After JWYPFS and DEX treatment, inflammatory cells in BALF were significantly reduced (Figure 2B). Furthermore, the results show that the numbers of Eos and macrophages in the OVA group increased significantly (Figures 2C,D). Compared with the OVA group, the JWYPFS-treated asthmatic mice had significantly fewer inflammatory cells, especially in the JWYPFS 4.0 group (high-dose group).

### JWYPFS Reduced Airway Hyperresponsiveness in OVA-Induced Asthma Mice

To evaluate the role of JWYPFs in OVA-induced airway hyperresponsiveness, airway resistance to atomized PBS or methylcholine was measured 48 h after the final OVA challenge. There were no significant differences between the six groups at baseline. Airway reactivity was significantly enhanced and RL was elevated in OVA-induced asthmatic mice. Administration of JWYPFS at 1.0, 2.0, or 4.0 g/kg reduced the airway hyperresponsiveness induced by methylcholine. DEX also significantly inhibited airway hyperresponsiveness, and the attenuation effect of JWYPFS on airway resistance was slightly weaker than that of dexamethasone (Figures 2E,F).

### JWYPFS Alleviated Inflammatory Cell Infiltration and Mucus Production in Mouse Lung Tissues

**FIGURE 3:**
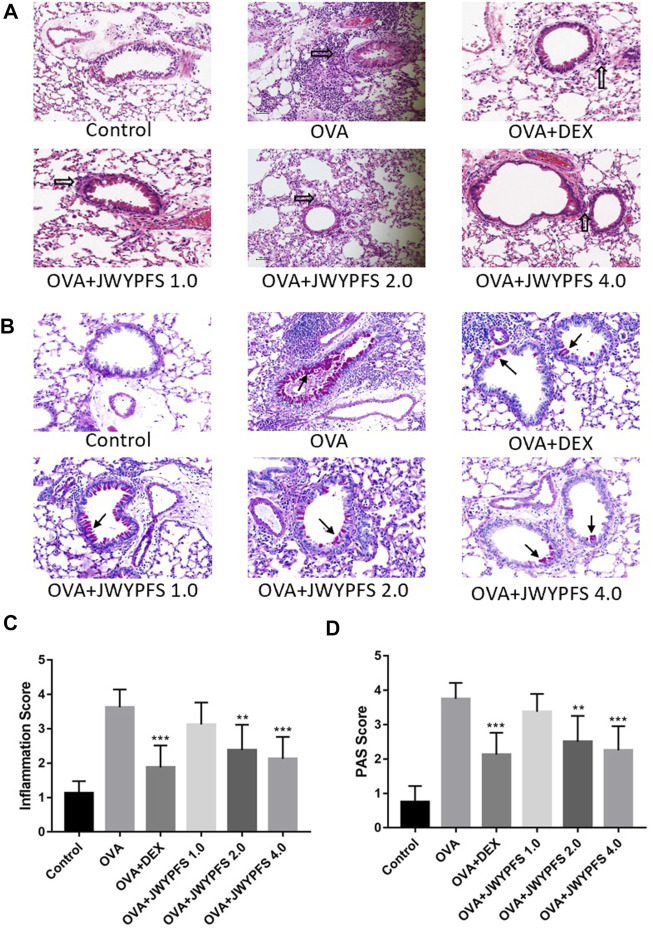
The effect of JWYPFS on inflammatory cells and mucus production in lung tissue. **(A)** Infiltration of inflammatory cells in the lung tissue as indicated by HE staining (×200). Black blank arrows indicate inflammatory cells; **(B)** Mucus secretion in lung tissue as indicated by PAS (×200). Black solid arrows indicate PAS-positive goblet cells; **(C)** Perivascular and peri-bronchial inflammation, scored as described in Materials and Methods. **(D)** A semi-quantitative analysis for the PAS-positive bronchi scoring. Data are shown as mean ± SD. *p < 0.05, **p < 0.01, ***p < 0.001, compared with OVA group.

HE staining was used to measure the inhibitory effects of JWYPFS on inflammation in lung tissue. As shown in Figure 3A, the bronchi and alveolar structure of the control mice was clear. OVA-group mice had a significant infiltration of inflammatory cells into peribronchiolar and perivascular connective tissues. JWYPFS treatment remarkably reduced the inflammatory cell infiltration (Figure 3C). To determine if JWYPFS alleviates mucus production in asthmatic mice, PAS staining was carried out on the lung sections for analysis. The OVA group had an obvious increase in mucus hypersecretion and proliferation of goblet cells. In contrast, the JWYPFS treatment group had significantly decreased mucus secretion, similar to the DEX group (Figure 3).

### JWYPFS Inhibited ILC2s in Lung Tissue of Asthmatic Mice

**FIGURE 4:**
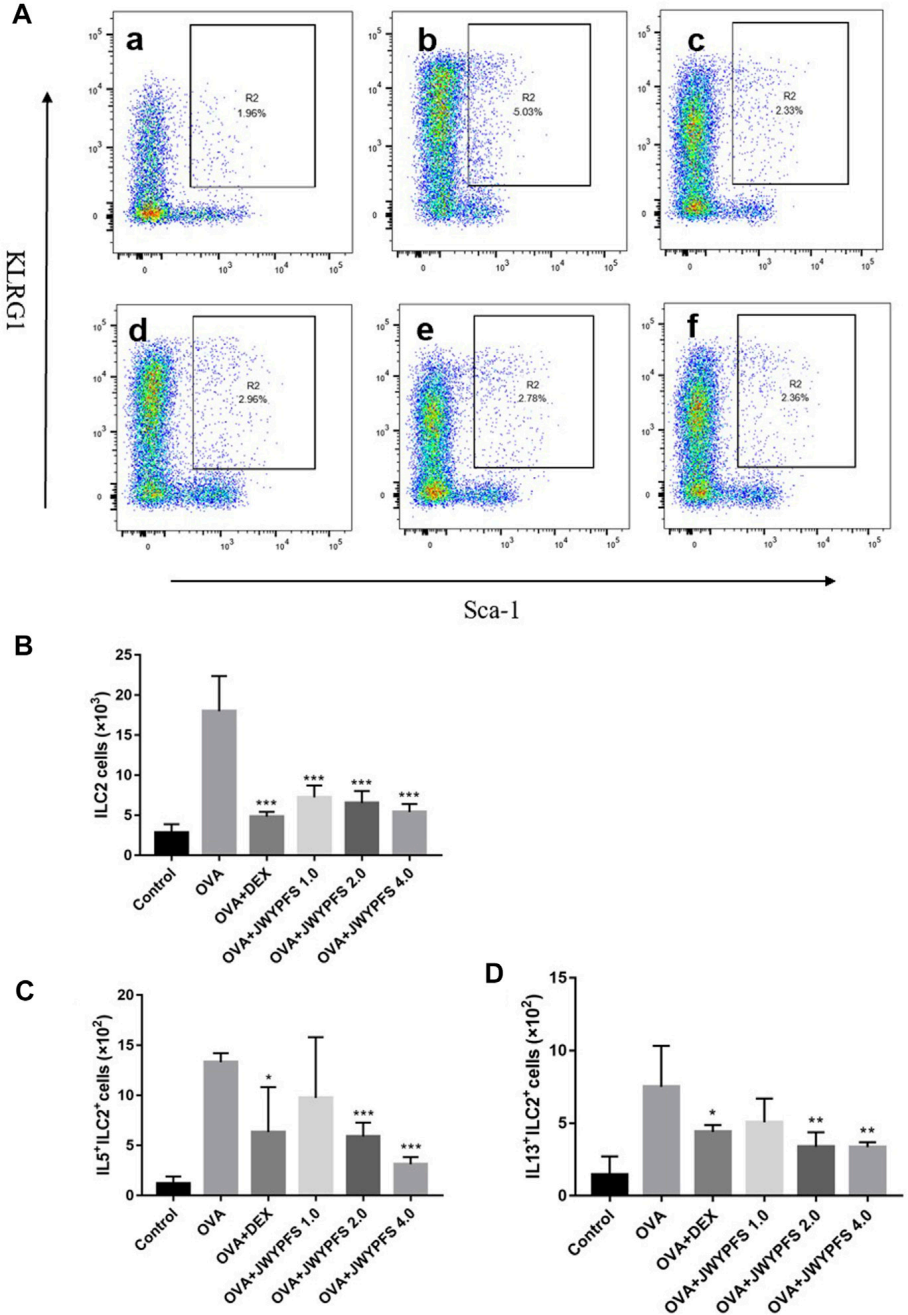
The effect of JWYPFS on ILC2s in lung tissue. **(A)** Flow cytometric analysis of the percentage of CD45^+^Lineage^−^Sca-1^+^KLRG1^+^ ILC2s in each group. a: control; b: OVA; c: OVA + DEX; d: OVA + JWYPFS 1.0; e: OVA + JWYPFS 2.0; f: OVA + JWYPFS 4.0. **(B)** Cell numbers of ILC2s in lungs. **(C)** IL5^+^ILC2s in lungs. **(D)** IL13^+^ILC2s in lungs. Data are shown as mean ± SD. *p < 0.05, **p < 0.01, ***p < 0.001, compared with OVA group.

Since ILC2s play an important role in regulation of the type 2 inflammatory response, ILC2s-targeted therapy has been considered as an attractive strategy for the treatment of allergic asthma in the last decade. In our study, the ILC2s number increased from (2.82 ± 1.07×10^3^) in the control group to (18.00 ± 4.39×10^3^) in OVA group. In contrast, oral administration of DEX and JWYPFS clearly inhibited the up-regulation of ILC2s. JWYPFS treatment reduced the amount of ILC2s, and the effectiveness increased with increasing dose (Figure 4B). Consistent with this, the data indicates that the proportion of ILC2s cells was markedly increased in the OVA group, while after JWYPFS administration the proportion of ILC2s cells markedly decreased in CD45^+^ Lineage^−^ cells (Figure 4A). Next, we examined the expression of type 2 cytokines by ILC2s. The OVA group had greater numbers of IL-5^+^ and IL-13^+^ ILC2s compared with the control group. A reduction of IL-5^+^ and IL-13^+^ ILC2s was observed in all mice treated with JWYPFS and DEX, as compared with the OVA group (Figure 4). Due to Th2 cells also express type 2 cytokines, Th2 cells were detected in the lung tissue of mice by flow cytometry. As shown in Supplementary Figure S1A,B, the OVA group had greater number and proportion of Th2 cells compared with the control group (*p*<0.000), while JWYPFS had no inhibitory effect on Th2 cells. At the same time, we further examined the IL-5 and IL-13 expression via Th2 cells by flow cytometry, which showed that JWYPFS had no effect on the IL-5^+^Th2 and IL-13^+^Th2 cells (Supplementart Figure S1C,D).

### JWYPFS Decreased ILC2s Cytokines and Transcription Factors in Lung Homogenate

**FIGURE 5:**
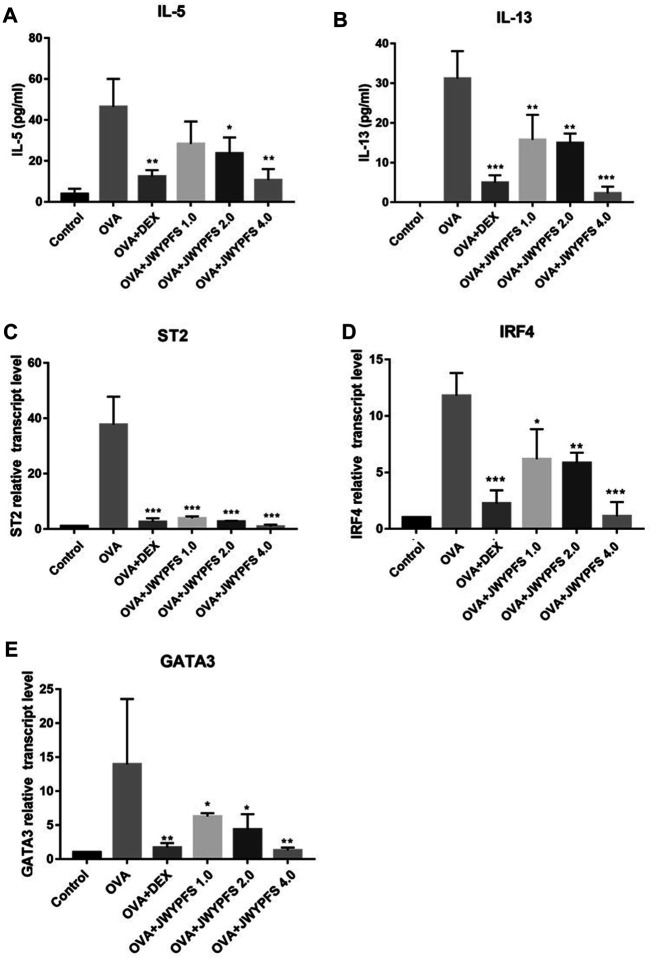
The effect of JWYPFS on ILC2s cytokine expression and related transcription factors in the lung. **(A)** The level of IL-5 in the lung by ELISA. **(B)** The level of IL-13 in the lung by ELISA. **(C)** Relative expression of ST2 mRNA in lung. **(D)** IRF4 mRNA expression in lung. **(E)** GATA3 mRNA expression in lung. Data are shown as mean ± SD. *p < 0.05, **p < 0.01, ***p < 0.001, compared with OVA group.

The transcription factors interferon regulating factor 4 (IRF4) and GATA-binding protein 3 (GATA3) are known to promote the expression of ILC2s cell differentiation and function including IL-5 and IL-13 expression (Madouri et al., 2017). ELISA was used to detect expression differences in inflammatory molecules such as IL-5, IL-13 in the control group, OVA group, JWYPFS and DEX treatment groups. The results show that the expression of IL-5 and IL-13 in the OVA group mice was significantly increased when compared to the control group, and these increases were reversed after the treatment with JWYPFS or DEX (Figure 5). Consistent with the cytokine data, the IL-33 receptor suppressor of tumorigenicity 2 (ST2), which is involved in ILC2s activation, and IRF4 and GATA3, transcription factors of ILC2s, were elevated in the OVA group. Nevertheless, administration of JWYPFS or DEX distinctly decreased the levels of ST2 (Figure 5C), IRF4 (Figure 5D) and GATA3 (Figure 5E) as compared with OVA-sensitized mice.

### JWYPFS Suppressed IL-33-Stimulated Enhancement of ILC2s Cytokines and Inhibited ILC2s Transcription Factor Expression *in vitro*

**FIGURE 6:**
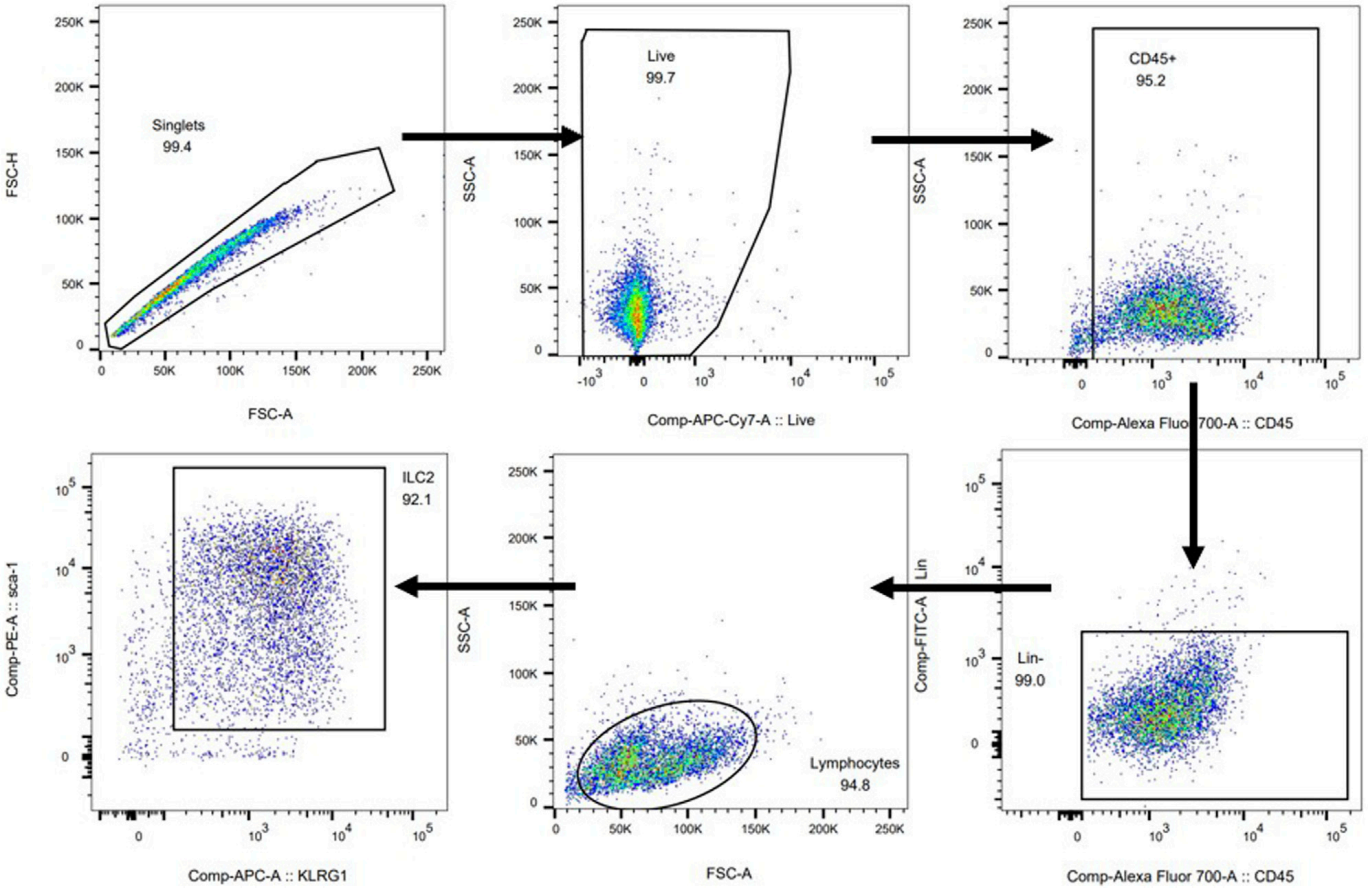
Cell sorting protocol and purity of ILC2s.

**FIGURE 7:**
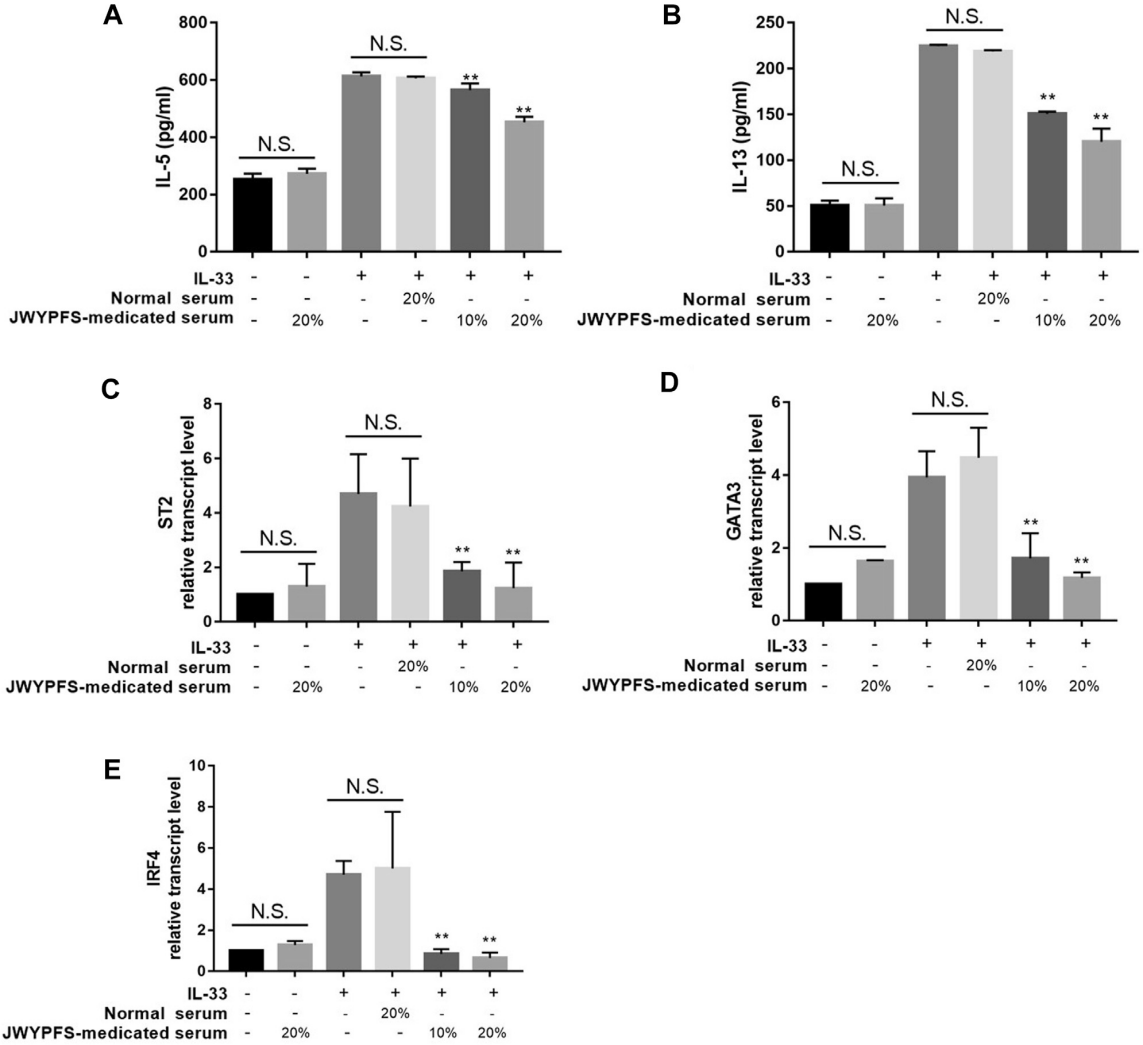
The effect of JWYPFS on type 2 cytokines and transcription factor expression in IL-33-stimulated ILC2s *in vitro*. **(A)** IL-5 in culture medium. **(B)** IL-13 in culture medium. **(C)** ST2 mRNA expression of ILC2s. **(D)** GATA3 mRNA expression. **(E)** IRF4 mRNA expression in ILC2s. Data are shown as mean ± SD. N.S. for not significant, *p < 0.05, **p < 0.01, compared with the IL-33 combined normal serum treatment group.

To further verify potential mechanisms of JWYPFS in ILC2s, we selected ILC2s from the lung tissue of mice (Figure 6) and intervened with JWYPFS-medicated serum. We found that cytokine production of IL-5 and IL-13 was significantly increased after IL-33 stimulation for 4 h. The IL-33 stimulation of ILC2s with normal serum treatment group was designated as the control group in this experiment. Results show that different doses of JWYPFS-medicated serum were able to interfere with IL-5 and IL-13 secretion (Figures 7A,B). Similarly, JWYPFS treatment significantly decreased the levels of the IL-33 receptor, ST2 (Figure 7C). To further investigate whether the effects JWYPFS on ILC2s were transcriptionally regulated, ILC2s transcription factor GATA3 and IRF4 were measured by RT-PCR. ILC2s were treated with IL-33 with or without JWYPFS-medicated serum. As shown in Figures 7D,E, compared to the control group, JWYPFS-medicated serum markedly inhibited GATA3 and IRF4 mRNA expression (Figures 7D,E).

## Discussion

Asthma is a complex and heterogeneous respiratory disease characterized by airway inflammation, airway hyperresponsiveness, mucus hypersecretion and restricted reversible airflow, resulting in wheezing, shortness of breath, chest tightness, cough and other clinical symptoms (Gauthier et al., 2015). The therapy for asthma has made progress with the development of ICS, beta2-agonist and bronchodilators, but not every asthma patient responds well to the current therapeutic strategy. Therefore, it is useful to seek more comprehensive strategies to prevent and reverse asthma, including complementary and alternative medicine (Liang et al., 2014). TCM has a long history of human use with a unique theoretical system and is popular in China due to its efficacy and few adverse effects. Based syndrome differentiation, TCM has distinct advantages in treating chronic respiratory diseases. Clinical and experimental studies have confirmed that TCM can improve asthma symptoms, reduce airway inflammation, inhibit airway hyperresponsiveness, and ameliorate airway remodeling (Jin et al., 2014; Zhang et al., 2016; Jiang et al., 2018; Cheng et al., 2019).

YPFS is an effective classic TCM prescription for asthma therapy. According to a clinical study, YPFS can significantly decrease symptoms and improve pulmonary ventilation in patients with chronic persistent asthma (Wang et al., 2020). Another study showed that YPFS may inhibit airway inflammation and reduce airway hyperresponsiveness by reducing IgE levels, modulating T lymphocyte subsets, and enhancing immune function in asthmatic mice (Wang et al., 2016). In addition, YPFS reduced the inflammatory response of asthmatic mice by inhibiting the NOD-like receptor family pyrin domain-containing 3 (NLRP3) inflammasome (Liu et al., 2017). Huangqi-Fangfeng, the main components of YPFS, inhibited airway remodeling by regulating epithelial-derived transforming growth factor beta-1 (TGF-β1) in a house dust mite (HDM)-induced allergic asthma mouse model (Yao et al., 2019). According to TCM theory, *Glycyrrhiza uralensis* Fisch. ex DC., *Schisandra chinensis* (Turcz.) Baill., *Rubus chingii* Hu, *Cullen corylifolium* (L.) Medik., *Aconitum carmichaeli* Debeaux, *Vitex negundo* L., and *Imperata cylindrica* (L.) P. Beauv. were added to the YPFS prescription to yield JWYPFS to enhance treatment outcomes. Although numerous studies have confirmed the benefit of JWYPFS in asthma, it is not clear whether JWYPFS has a therapeutic effect on the type 2 inflammation response, especially the cytokines produced by ILC2s.

Airway inflammation is an important characteristic of asthma. Inflammatory cells, such as Eos and lymphocytes, accumulate in the airways and release a series of pro-inflammatory products and cytotoxic substances, causing edema, mucus hypersecretion, tissue damage and airway hyperreactivity, which is an important link in the pathogenesis of allergic asthma (Hogan et al., 2008). In our study, a mouse model of OVA-induced allergic asthma was employed to assess the effect of JWYPFS on the suppression airway inflammation. We found that the oral administration of JWYPFS inhibited the elevation of inflammatory cells in BALF, particularly the Eos. Consistent with the result with BALF, HE pathological analysis suggested that JWYPFS alleviated the infiltration of inflammatory cells in the lungs of asthmatic mice. Moreover, JWYPFS also suppressed mucus secretion in the lung.

Previous studies have elucidated that Th2-type immune responses contribute to allergic eosinophilic airway inflammation as the underlying mechanism of asthma (Wills-Karp et al., 1998; Kips et al., 2001; Deo et al., 2010). In recent decades, researchers have found that ILC2s also produce type 2 cytokines and play a key role in the pathogenesis and control of asthma (Jia et al., 2016; Yu et al., 2018; Salter et al., 2019). The literature has demonstrated that when epithelial cells contact allergens, the cells release alarmins, such as IL-33 and TSLP. Subsequently, alarmins activate ILC2s to produce Th2 type cytokines, and Eos is recruited to stimulate airway inflammatory responses (Klein Wolterink et al., 2012; Johansson et al., 2018; Jonckheere et al., 2019; Li et al., 2019). Another clinical study has reported that compared with mild asthma and a nonatopic control group, severe asthma patients showed increased type 2 cytokine-producing ILC2s in sputum. Moreover, after oral corticosteroid therapy, asthma patient with increased ILC2s are more likely to develop steroid resistance (Liu et al., 2018). This suggests that targeting ILC2s may provide new therapeutic strategies for asthma treatment. To analyze the inhibitory effects of JWYPFS on ILC2s, we used flow cytometry to detect ILC2s, and found that both the cell count and proportion of ILC2s decreased after JWYPFS treatment. The number of ILC2s expressing IL-5 and IL-13 also decreased. JWYPFS reduced the levels of the transcription factors GATA3 and IRF4 of ILC2s, the receptor for the activation factor IL-33, ST2 and type 2 cytokines such as IL-5, and IL-13. In order to further verify the effect of JWYPFS on ILC2s-mediated airway inflammation, ILC2s were isolated and treated with JWYPFS. JWYPFS inhibited type 2 cytokine secretion in IL-33-induced ILC2s. Furthermore, ILC2s transcription factors GATA3 and IRF4 were also dramatically decreased by JWYPFS *in vitro*. One of a potential limitation of this study is that neither GATA3 or IRF4 has been studied at protein level, particularly in the nuclear extract of the ILC2s. However, the amounts of ILC2s were limited, thus it is very difficult to quantify the protein levels of both GATA3 and IRF4 in ILC2s. This is also the difficulty of studies related to ILC2s. Our results show that the anti-inflammatory effect of JWYPFS is slightly weaker than that of DEX. However, JWYPFS is safer than DEX and has fewer adverse effects. JWYPFS can be taken orally for a prolonged period and can be used in the clinical treatment of asthma. It was reported that patients with severe asthma showed increased production of ILC2s cytokines in sputum, compared to those with mild asthma and healthy controls, and asthmatic patients with increased ILC2s often develop steroid resistance after systemic glucocorticoid therapy (Smith et al., 2016). Our results show that JWYPFS has a beneficial effect on ILC2, which suggests that it is possible to combine the use of JWYPFS with reduced steroid treatment to decrease the possibility of development of steroid resistance, while providing good control of the disease. Pregnant women and children should use JWYPFS under the guidance of a doctor. We have not find any drugs can be used in combination to enhance the efficacy yet, however, according to our results, asthma patients who are prone to develop resistance to steroid drugs can combined with JWYPFS to reduced steroid treatment. As a mixture of ten herbal extracts, JWYPFS contains multiple constituents. Thus, it is very difficult to identify the most active one(s) in this herbal medicine with regard to the symptoms of asthma. In the future, we will try to identify the key bioactive constituents in JWYPFS by using a panel of modern techniques and related bioactivity assays.

In summary, the treatment with JWYPFS alleviated airway inflammation with inhibition of inflammatory cells, Eos infiltration, and type 2 cytokines, and reduced the percentage and cell number of ILC2s in mice with OVA-induced asthma. In addition, type 2 cytokines secreted by IL-33-induced ILC2s were also effectively suppressed by JWYPFS. Our findings provide a possible route for the clinical application of JWYPFS in the treatment of allergic asthma.

## Conclusion

In summary, our results clearly demonstrate that JWYPFS inhibits the type 2 response mediated by ILC2s and reduces the airway inflammation of allergic asthma. Therefore, JWYPFS might be a beneficial therapeutic drug against airway inflammation in allergic asthma. However, JWYPFS contains a variety of chemical components, and further research is needed to explore the anti-inflammatory mechanisms of the main molecular components.

## Data Availability

The datasets presented in this article are not readily available because Since this project is funded by the National Natural Science Foundation of China and has not yet been completed, it is limited that apply to the dataset. Requests to access the datasets should be directed to Dr._luzh@shutcm.edu.cn.

## Supplementary Material

Supplementary Figure 1 The effect of JWYPFS on Th2 in lung tissue. **(A)** Flow cytometric analysis of the percentage of CD45^+^CD4^+^IL-4^+^ Th2 in each group. a: control; b: OVA; c: OVA + DEX; d: OVA + JWYPFS 1.0; e: OVA + JWYPFS 2.0; f: OVA + JWYPFS 4.0. **(B)** Cell numbers of Th2 in lungs. **(C)** IL5^+^Th2 in lungs. **(D)** IL13^+^Th2 in lungs. Data are shown as mean ± SD. ^###^ *P* < 0.001, compared with control group; **P* < 0.05, ***P* < 0.01, ****P* < 0.001, compared with OVA group.

## Abbreviations

ILC2s, group 2 innate lymphoid cells; JWYPFS, Jia-Wei-Yu-Ping-Feng-San; YPFS, Yu-Ping-Feng-San; OVA, ovalbumin; BALF, bronchoalveolar lavage fluid; GATA3, GATA-binding protein 3; IRF4, interferon regulating factor 4; Th2, T helper two; IL, interleukin; Eos, eosinophils; ILCs, innate lymphoid cells; TSLP, thymic stromal lymphopoietin; ICS, Inhaled corticosteroids; TCM, Traditional Chinese medicine; HPLC, High performance liquid chromatography; DEX, dexamethasone; PBS, phosphate-buffered saline; HE, hematoxylin-eosin; PAS, periodic acid Schiff; ST2, suppressor of tumorigenicity two; NLRP3, NOD-like receptor family pyrin domain-containing 3; TGF-β1, transforming growth factor beta-1; HDM, house dust mite.
