# Supplementary figures and images for: Jia-Wei-Yu-Ping-Feng-San Attenuates Group 2 Innate Lymphoid Cell-Mediated Airway Inflammation in Allergic Asthma
Source: Front Pharmacol. 2021 Jul 9;12:703724. doi: 10.3389/fphar.2021.703724 (PMC8299004; doi:10.3389/fphar.2021.703724)

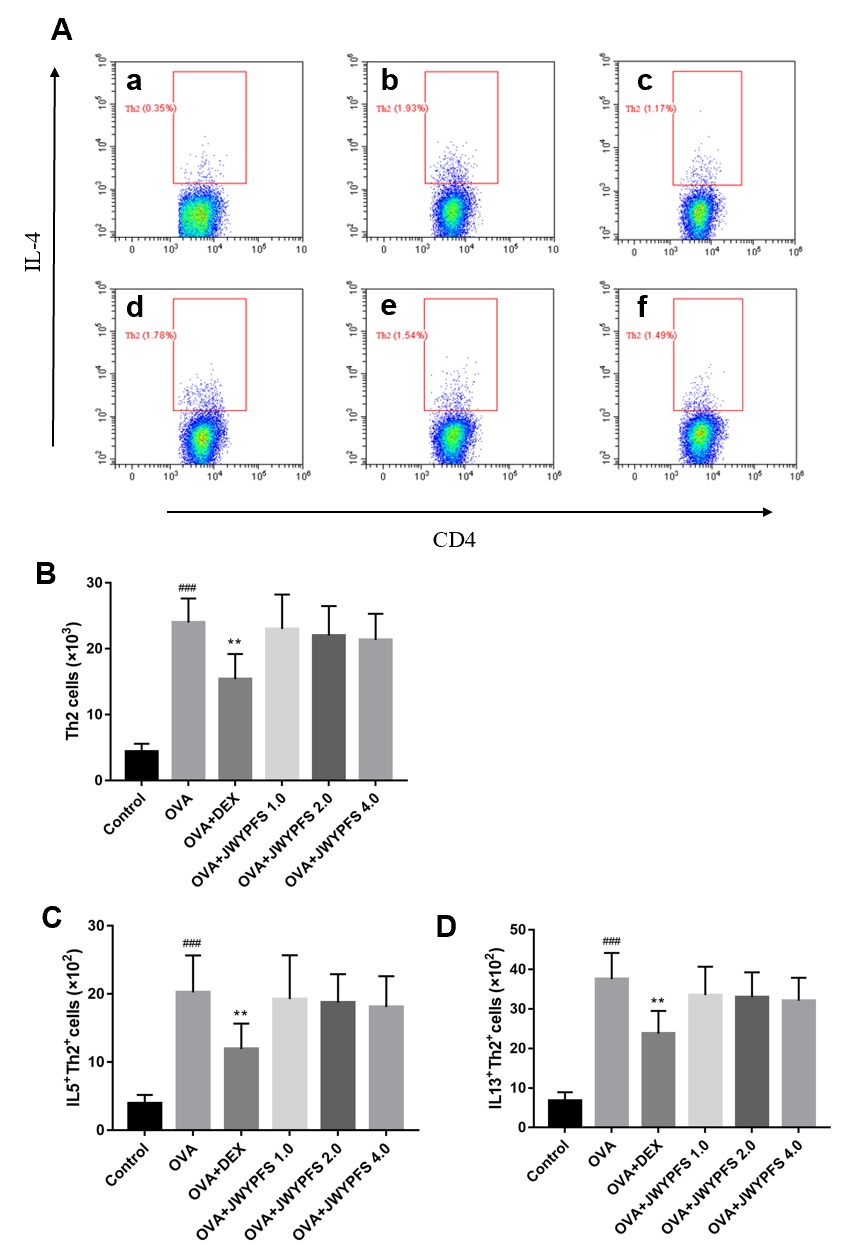

Supplement: Supplementary file 1 [file Image1.JPEG]
